# Family Planning Among Canadian Plastic Surgeons and Trainees

**DOI:** 10.1177/22925503221151187

**Published:** 2023-01-17

**Authors:** Austin Kemp, Katie Garland, Elise Graham, Andrew Simpson, Caitlin Symonette

**Affiliations:** 1 Schulich School of Medicine and Dentistry, 6221 Western University, London, ON, Canada; 2 Division of Plastic and Reconstructive Surgery, 6221 Western University, London, ON, Canada; 3 Division of Otolaryngology, 6221 Western University, London, ON, Canada

**Keywords:** plastic surgery, family planning, maternity leave, paternity leave, breastfeeding

## Abstract

**Introduction:** Despite increasing advocacy for family leave policies, few studies have described the current landscape and attitudes around family planning in Canadian plastic surgery. The purpose of this study was to survey Canadian plastic surgeons and trainees to examine their experience with family planning, parental leave, and breastfeeding. **Methods:** An anonymized survey was distributed to all members of the Canadian Society of Plastic Surgeons and all Canadian Plastic Surgery residents through their program administrators. Survey responses were recorded and analyzed through a customized REDCap™ database. Results were reported using descriptive statistics. **Results:** A total of 87 plastic surgeons and trainees completed the surgery. We found 72.3% of respondents had children; 67.8% felt their colleagues were supportive of parental leave; 45.6% felt that financial concerns affected their decision to take parental leave; 61.6% felt that their career did not influence the number of children they chose to have; 21.0% accessed fertility services and 9.8% used assisted-reproductive technologies; 80% of respondents who breastfeed felt they did not have enough time to pump at work, however, 79% did not experience any discrimination or criticism for pumping at work. **Conclusion:** Canadian plastic surgeons most often have children after completing training and choose to take shorter parental leaves as their careers progress. Parental leave and breastfeeding practices in the workplace are reported to have increased support from colleagues compared to previous literature. However, Canadian plastic surgeons continue to struggle with infertility and seek fertility services at rates higher than the general population.

## Résumé

**Introduction :** En dépit d’un plaidoyer croissant pour les politiques de congé familial, peu d’études ont décrit le paysage actuel et les attitudes concernant le planning familial dans le monde de la chirurgie plastique au Canada. L’objectif de cette étude était de mener une enquête évaluant les attitudes et expériences du personnel et des résidents de chirurgie plastique au Canada concernant le planning familial au cours de leur carrière. **Méthodes :** Une enquête anonyme a été distribuée à tous les membres de la Société canadienne des chirurgiens plasticiens, en plus de tous les résidents des programmes canadiens de chirurgie plastique par le biais des administrateurs de leurs programmes. Les réponses à l’enquête ont été consignées et analysées dans une base de données adaptée REDCap^MD^. **Résultats :** Un total de 87 chirurgiens plasticiens et stagiaires ont rempli le questionnaire. 72,3% des répondants avaient des enfants. 67,8% ont estimé que leurs collègues soutenaient les congés parentaux. 45,6% ont estimé que des préoccupations financières affectaient leur décision de prendre un congé parental. 61,6% ont estimé que leur carrière n’influençait pas le nombre d’enfants qu’ils choisissaient d’avoir. 21,0% ont eu accès à des services de fertilité et 9,8% ont utilisé des technologies de reproduction assistée. 80% des répondantes qui allaitaient estimaient qu’elles n’avaient pas assez de temps pour pomper leur lait au travail; toutefois, 79% n’ont éprouvé aucune discrimination ou critique pour le faire pendant les heures de travail. **Conclusion :** Les chirurgiens plasticiens canadiens ont le plus souvent des enfants après la fin de leur formation et choisissent de prendre des congés parentaux plus courts au fur et à mesure de l progression de leur carrière. Les congés parentaux et les pratiques d’allaitement sur les lieux de travail bénéficient d’un plus grand soutien de la part des collègues, comparativement à des publications antérieures. Cependant, les chirurgiens plasticiens canadiens continuent de lutter contre les problèmes d’infertilité et recherchent des services d’aide à la procréation à un taux supérieur à celui de la population générale.

## Introduction

Pregnancy and family planning are challenging for medical trainees entering surgical disciplines, as well as practicing surgeons.^1^ Despite advocacy efforts for pregnancy and family leave policies, family planning remains a polarizing subject. Plastic surgery training is demanding and occurs over a 5 to 6 year period, which often overlaps trainees’ childbearing years. Many trainees pursue advanced graduate training (Masters/Postdoctoral degrees), which can add an additional 2 to 4 years before completion. Trainees carry large sums of debt into practice, which places financial burden when one transitions to practice. To effect positive change for future generations, it is important to understand which factors are impacting family planning for staff surgeons and trainees. This study aims to examine pregnancy, family planning, parental leave, and breastfeeding among Canadian plastic surgeons.

Plastic surgery trainees must consider the impact family planning has on their training, ability to balance work and parenthood, potential pregnancy complications, and the impact on their colleagues. A 2019 survey of Canadian plastic surgery residents reported that 72% did not want to have children during residency for several reasons, with the most common being an unwillingness to delay training.^2^ They also found that of the residents and recent graduates who did become pregnant, 42% experienced complications, more than double that of the general population.^3^ Furthermore, plastic surgery training programs are historically small and accept 1 to 4 trainees per cohort. As such, the same study found almost 60% of residents reported that their parental leave negatively affected the workload of others.^2^ Due to the inherent size and work structure of plastic surgery programs, parental leave puts an added workload onto the remaining residents. A study from the American Society of Plastic Surgeons found that 72.6% of women and 39.2% of men delayed starting a family due to training demands.^4^ Another survey reported that 71.0% of female trainees and 51.0% of male trainees reported negative stigma attached to pregnancy.^5^ For trainees that choose to start a family during training, assisted reproductive technologies (ART) were used by 20% of trainees. Additionally, 62% of females and 51% of males reported dissatisfaction with their parental leave.^5^

In Canadian literature, data looking at family planning and pregnancy among practicing plastic surgeons is limited. In addition to pressures from work schedules and training demands, time is a critical factor to consider. It is well established that rates of infertility and pregnancy related complications increase significantly with deferred childbirth.^6^ A survey of female surgeons across multiple disciplines showed a 3 times higher infertility rate compared to the general public and a higher likelihood of seeking ART.^7^ In Canada, the average age of first-time mothers is 29.2 years, whereas the average age of trainees completing their surgical residency is 33.3 years.^8,9^ Consequently, surgeons have been shown to have fewer biological children and be closer to advanced parental ages at the time of their first pregnancy compared to the general population.^5,10^ Practicing plastic surgeons also face financial pressures as they possess financial responsibilities, such as debt and overhead, that are often not covered by the supports available. This impacts the duration of parental leave practicing surgeons take, as longer periods away from practice have greater financial implications.

With a turn towards increasing awareness and discussion about family planning in surgery, it is important to first understand the current landscape surrounding family planning and pregnancy in the Canadian plastic surgery population. This study aims to examine the current attitudes and experiences of Canadian plastic surgeons and trainees with respect to family planning, pregnancy, parental leave, and breastfeeding over the course of their careers. The data collected in this study is essential for identifying areas of concern, while improving experiences and support for plastic surgeons who look to start families.

## Methods

A literature search was performed to assess previous questionnaires surveying pregnancy and parental leave among physicians in Canada and the United States. A comprehensive survey was adapted from Lawlor et al^11^ Selected questions were reviewed by the research team. The survey used branching logic and was designed to cover questions related to demographics, family planning and fertility, parental leave, and breastfeeding. Respondents answered 30 to 40 questions. The survey was available in French and English and contained a letter of information and consent. The introductory page explained that participation was voluntary, outlined the risks involved, and consent was given when respondents submitted their survey. Any question could be skipped as none were mandatory and respondents could exit the survey at any time.

Following approval by our Institutional Review Ethics Board (IRB 119020), the anonymized survey was sent to all plastic surgeons registered with the Canadian Society of Plastic Surgeons and each Canadian plastic surgery residency program to be distributed among trainees. This study cohort targets training and practicing plastic surgeons in Canada. An additional reminder email was sent to each subset. The survey was open for 5 months. Survey responses were recorded through a secure customized REDCap™ electronic database (Vanderbilt University, Nashville, TN, USA). Results were reported using descriptive statistics.

## Results

### Demographics

**Figure 1.**
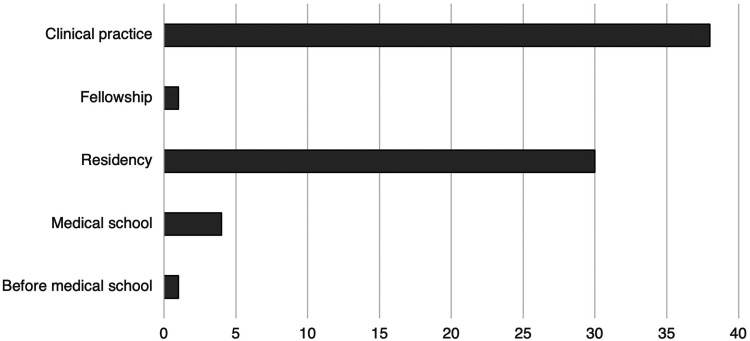
Distribution of when Canadian plastic surgeons and trainees had children in throughout their careers. *Data from respondents who had children.

There were 87 respondents. Sixty-three staff, 17 residents, 2 fellows, and 1 retired surgeon completed the survey. Four did not specify level of training. Response rate was estimated to be 15%. Table 1 demonstrates the demographic characteristics of respondents. Forty-one (49.4%) identified as cisgender male, 39 (47.0%) as cisgender female, 2 (2.40%) as agender, and 1 (1.20%) as “other.” Median age of respondents was 41 years (IQR 34.5-52). Sixty (72.3%) respondents had children (median number 3 [IQR 2-3]) and 12 (13.8%) respondents were planning on having children in the future. The median age of respondents at the birth of their first child was 32 years (IQR 29-35). Figure 1 demonstrates the distribution of when respondents had children.

**Table 1.** Demographic Characteristics of Survey Respondents.

Characteristic	No. (%) of respondents	No. in years
Sex		
Male	41 (49.4)	
Female	39 (47.0)	
Agender	2 (2.40)	
Other	1 (1.20)	
Level of training		
Resident	17 (20.5)	
Staff	63 (75.9)	
Fellow	2 (2.4)	
Retired	1 (1.20)	
Practice setting		
Academic	32 (38.6)	
Community	20 (24.1)	
Mixed	14 (16.9)	
Resident/Fellow	17 (20.5)	
Median age		41 (IQR 34.5-52)
Median age at first child		32 (IQR 29-35)
Median no. of children		3 (IQR 2-3)
Marital status		
Married/ common-law	69 (83.1)	
Single	6 (7.20)	
Cohabitating	4 (4.80)	
Divorced	3 (3.60)	
Other	1 (1.20)	

### Family Planning

Forty-five (54.2%) respondents reported to agree/somewhat agree that their department leaders were supportive of residents starting families; whereas 12 (14.5%) were neutral and 17 (20.5%) disagreed/somewhat disagreed.

Twenty-eight (34.1%) respondents agreed/somewhat agreed with the statement that training/practice influenced their decision to have children; 55 (67.0%) felt it influenced their decision about when to have children, and 20 (24.4%) agreed it influenced their ability to have children. The majority (61.6%) of respondents felt that training/practice did not influence the number of children they chose to have, but 13 (21.6%) felt that training influenced their ability to conceive as many children as they wished to have.

Seventeen (21.0%) respondents accessed fertility services and 8 (9.80%) used ART. Twenty-five (41.0%) respondents had a miscarriage. Of the 41.0% who reported having a miscarriage, the average number of miscarriages was 2.1 (SD 3.07). Twenty (24.4%) respondents agreed/somewhat agreed that they were concerned about their future fertility. Twenty-four (29.6%) agreed/somewhat agreed they had concerns about future parental leave.

### Maternity Leave/Paternity Leave

Of the respondents who had children, 31 (52.5%) took parental leave. Of the respondents who took maternity leave, 32 (47.7%) respondents reported to agree/somewhat agree that the department was supportive of their leave. Whereas, of the respondents who took paternity leave, 13 (21.0%) felt the department was supportive of their leave. Twenty-seven (32.5%) respondents disagreed/somewhat disagreed that the department was supportive of paternity leave. Of the 13 that felt the department was supportive of paternity leave, 9 (69.2%) were males and of the 27 that disagreed/somewhat disagreed that the department was supportive of paternity leave, 15 (55.6%) were males. Twenty-one (67.8%) respondents reported to feel their colleagues were supportive of maternity/paternity leave.

**Figure 2.**
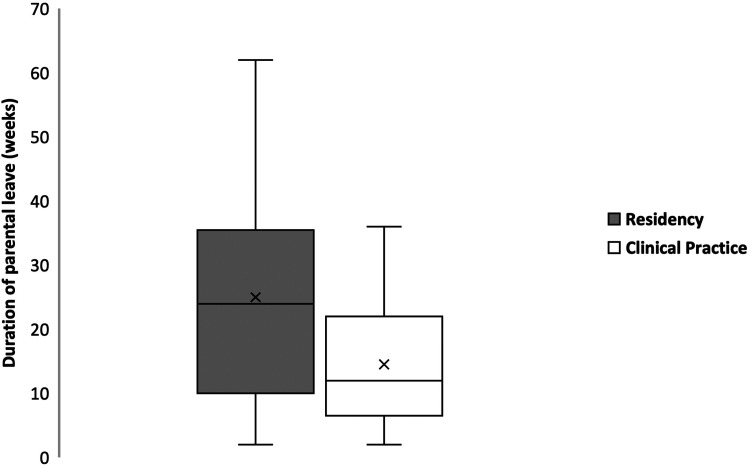
Box plot comparing duration of parental leave in weeks taken for leave by Canadian plastic surgeons and trainees during each stage of training (*x* represents the mean). *Data from respondents who had children.

On average, respondents stopped overnight or weekend call at 33.5 weeks gestational age (SD 5.99). Thirty-one (52.5%) respondents took parental leave; Figure 2 describes the average number of weeks taken for leave during each stage of training. Twenty-four (52.1%) respondents felt that leave had little to no effect on their surgical skills, and 25 (53.2%) felt parental leave had little to no effect on their future opportunities. Twenty-six (45.6%) felt that financial concerns moderately to strongly affected their decision to take leave and 12 (21.0%) felt difficulty finding coverage for their practice affected their decision.

### Breastfeeding

Of the childbearing females who participated, 25 (89.3%) respondents planned on breast feeding their children, 9 (36.0%) pumped breast milk at work as a resident and 11 (55.0%) pumped as a staff. Sixteen (80.0%) respondents felt they did not have enough time to pump at work and 15 (75.0%) of respondents denied adequate space to pump at work. Furthermore, 14 (70.0%) respondents felt they did not have adequate storage for pumped breast milk at work. Eleven (55.0%) respondents felt they did not meet their breastfeeding goals. Most respondents (79.0%) did not experience discrimination or criticism for pumping at work.

## Discussion

Our results suggest that the plastic surgery environment in Canada has seen progress when looking at pregnancy, parental leave, and breastfeeding, however, highlights continued room for improvements.^2^ The average age at first child was 32.1, 3 years older than the average maternal age at first pregnancy for the general Canadian population.^8^ This is in keeping with the average age at completion of surgical training. However, this is not unique to plastic surgery as it is similar to women of higher levels of education and other surgical training programs.^9,12^ Female fertility drops nearly in half from the early 20s to the late 30s.^13^ Studies have also shown female surgeons birth their first child even later if ART are used.^7,14^ We found one-fifth of respondents to have accessed fertility treatments and less than 10% used ART. Having said this, our results still suggest an increase in using ART compared to the general Canadian population.^15^

A 1995 study looking at family planning in plastic surgery reported 36% of program directors in the United States actively discouraged pregnancy among residents and 45% felt that pregnancy adversely affected the program.^16^ A recent Canadian study found over half of respondents heard negative comments from staff or residents related to pregnancy during training.^2^ The results from our study show over half of respondents felt their department leads were supportive of residents starting families during training. However, only 47.7% and 21.0% felt their department was supportive of maternity leave and paternity leave, respectively. Nonetheless, most respondents in our study did not feel that training influenced the number of children they chose to have, rather the timing of childbirth.

Despite longstanding impressions that parental leave negatively effects a physician's career, studied benefits of parental leave include lower infant mortality and morbidity, improved maternal health outcomes, lower divorce rates, and improved child–parent relationships.^17–20^ With approximately 10 to 20 plastic surgery trainees per residency program, parental leave combined with current on-call structures, place an added workload on the remaining residents compared to larger programs.^2,21^ Trainees wishing to start a family during residency must balance their parenting needs with training requirements. Yet, results showed that over half of respondents felt leave had little to no effect on their surgical skills and would not significantly impact their future opportunities. These findings are consistent with a 2020 study, showing that almost 70% of residents disagreed that a resident would graduate with less experience if they took parental leave.^2^ The American Board of Obstetrics and Gynecology spearheaded flexible leave schedules and found missed time due to parental leave had no significant impact on surgical experience.^22^

Furthermore, our study found almost half of respondents felt financial concerns and 20% felt difficulty finding coverage affected their decision to take leave. Over 45% of Canadian medical students graduate with at least $100 000 of debt, and residents often graduate paying off little debt.^23^ Practicing surgeons wishing to start or grow their family must consider the cost of living, clinical overhead, and maintaining debt payments while not actively billing. In Canada, financial support for parental leave is imposed by the provincial governments and medical associations. The Pregnancy and Parental Leave Benefit Program provides Ontario physicians with a maximum of $1300 weekly for 17 weeks, totaling a maximum of $22 100 if they meet the eligibility criteria.^24^ The Doctors of BC Parental Leave Program pays up to $1000 per week for 17 weeks.^25^ Balancing financial pressures on limited income is challenging. Our results showed that practicing plastic surgeons took on average 14.5 weeks parental leave, fewer than the allotted coverage based on province of practice. This could be attributed to financial considerations. Additionally, plastic surgery practices, especially in community settings are small teams of providers. Parental leave therefore requires colleagues to cover their service or hire locums, which can pose further financial implications. However, financial pressures are only one of several factors contributing to shorter parental leaves. In academic settings, active research participation may also contribute to shorter than allotted parental leave as these activities are not easily transferred. Few work models exist within plastic surgery that allow for alternative working patterns for better family planning and work–life balance. It is also important to acknowledge that plastic surgery nurtures a strong professional and personal identity within the workplace that may lead to earlier return to work.

In contrast, Canadian medical residents have protected parental leave though their respective provincial resident association. In Ontario, Professional Association of Residents of Ontario (PARO) offers 17 weeks of pregnancy leave prior to the due date, and up to 35 weeks of standard parental leave benefits. These benefits offer a combination of employment insurance and a supplemental income top up program, which increases income to 84% of regular weekly earnings.^26^ Similarly, the Professional Association of Resident of Alberta (PARA) offers 17 weeks leave time at 90% salary combined with EI benefits.^27^ As residents do not have the same financial pressures of running a practice, this benefit provides trainees with financial security throughout parental leave compared to practicing physicians. Our study found that plastic surgery residents took longer parental leave than their in-practice counterparts. Reasons why this difference exists may partially be explained by the financial benefits offered to each demographic.

Breastfeeding has significant benefits to both mother and child. Maternal benefits include reduction in post-partum complication and longer term benefits such as decreased risk of certain cancers, osteoporosis, cardiovascular diseases, and dementia.^28^ Fetal benefits are numerous and include improved survival and decreased rates of obesity, autoimmune conditions, infections, and poor health outcomes.^29^ Our results demonstrate that plastic surgeons and trainees have difficulties with meeting breastfeeding needs, however, do not feel discriminated against or criticized for breastfeeding at work. The former finding is consistent with much of the existing literature. Merchant et al^30^ found that Canadian general surgery residents felt being too busy, not having a place to pump milk, and feeling unsupported by staff and colleagues were major barriers to breastfeeding. Several studies have shown data consistent to ours expressing concerns around time or access constraints to lactation facilities to breastfeed, as well as ending breastfeeding earlier than desired and not meeting their breastfeeding needs.^5,10^ Throughout North American law, breastfeeding in public or at work is to be protected against discrimination and harassment.^31^ However, a recent 2018 survey demonstrated concern about derogatory comments about breastfeeding at work.^10^ Interestingly, our results suggest that almost 80% of respondents who breastfed did not experience discrimination or criticism in the workplace. These findings potentially represent a greater acceptance of breastfeeding in the workplace compared to previous. Reasons for this are likely numerous, however, an increase in the shared understanding of breastfeeding as a means for improved maternal and fetal health and increased advocacy around breastfeeding cannot be discounted.

It is challenging to create environments that concurrently optimize both childbearing and surgical education. Although our results suggest there may be less discrimination and more support around breastfeeding and family planning, further improvement is needed. Studies have proposed solutions to improve the family planning environment including flexible rotations schedules or research blocks so trainees can schedule rotations with lighter workloads during pregnancy, providing lactation facilities and adequate breaks to facilitate lactation at work, and providing on-site 24-hour childcare.^4,5^ Additional suggestions include trainee education around the impact of postponing childbearing on fertility, providing fertility counselling options such as oocyte preservation, mandating time-off call earlier in pregnancy, as well as improved parental leave benefits for staff. These modifiable changes need to be made at higher levels than plastic surgery departments. The Canadian Medical Association and provincial residency associations should spearhead advocacy for these changes at a national level. These efforts will increase job satisfaction and work life balance, while reducing burnout. Future research could build on our findings by looking at tangible changes such as the ones mentioned and quantifying the impact on childbearing, family planning, and parental leave throughout a surgeon's careers.

Limitations to this study are multifactorial and include untested survey reliability, and lack of full participation from Canadian Society of Plastic Surgeons membership and plastic surgery trainees. With lack of full participation, one must consider responder bias. Invitees who are passionate about creating change in this area or have had personal experiences with family planning are more likely to participate. Non-responders may have opinions, which differ greatly from survey participants.^32^ Thus, results may be skewed towards more negative opinions about family planning, breastfeeding, and parental leave and not reflect all plastic surgeons. We also recognize this survey does not address all issues relevant to this topic.

## Conclusion

Family planning among Canadian plastic surgeons and trainees is not broadly researched; however, it is directly relevant to the functioning of Canadian plastic surgery departments. Our results demonstrate Canadian plastic surgeons most often choose to have children after their training is complete and choose to take shorter parental leaves as their careers progress. We also found that this population continues to struggle with infertility and seek fertility services at rates higher than the general population. We highlight areas of improvement, including, more support from peers around starting families and taking parental leave during training, as well as less discrimination and criticism around breastfeeding in the workplace. However, a lack of support around parental leave from departments persists. Future studies can look at quantitative ways to identify modifiable factors to improve family planning and gender differences within Canadian plastic surgery. We hope this study will continue the conversation around family planning to improve the environment for future plastic surgeons.
